# Some Practical Thoughts on the Development of the Human Race and Obstetric Nursing

**Published:** 1897-03

**Authors:** R. R. Kime

**Affiliations:** Atlanta, Ga., Ex-President Atlanta Obstetrical Society; Ex-President Atlanta Society Medicine; Ex-Vice-President Tri-State Medical Society of Georgia, Alabama, and Tennessee


### SOME PRACTICAL THOUGHTS ON THE DEVELOPMENT OF THE HUMAN RACE AND OBSTETRIC NURSING.

BY R. R. KIME, M.D., ATLANTA, GA.,

Ex-President Atlanta Obstetrical Society; Ex-President Atlanta Society Medicine; Ex-Vice-President Tri-State Medical Society of Georgia, Alabama, and Tennessee.

There are primal forces of great importance in the development of the human race that should be taught and firmly impressed upon all parents. The principal factors are heredity, development, training, and association.

Many mental characteristics, physical defects, and tendencies to certain diseases are transmitted to the third and fourth generations, as taught by the Bible, science, and practical experience.

This being true, we can better understand why heredity plays such an important part in the improvement of the human race, yet development, training, and association are important factors which should be utilized.

Children are the reflex of parents in life's looking-glass. That reflection may be polished and improved or roughened and degraded at the will of the parents.

There is no question but that the child in utero is influenced by the mother during pregnancy. It is equally true that certain traits of character, mental and physical, are transmitted, whether they be elevating or degrading in their tendency.

Sowing of "wild oats" by the young is but the developing of vices and habits at a formative period that degrade true manhood and womanhood, thus lowering the standard from which the race must be propagated. Habits and traits of character thus formed are transmitted to the offspring. The use of opiates, whisky, and even tobacco, transmits influences to the child for which it is not so much to blame as the parent. The parent is doubly responsible when such vices and habits are formed simply as a gratification of desire for pleasure. Any true father should be willing to forego some of the gratifications of the appetite and transient pleasures of life for the sake of his offspring, humanity, and for the perpetuation of those nobler qualities and higher principles that characterize true manhood.

The human race is improved or degraded, physically, morally, intellectually and spiritually, at the will of the parents.

Heredity is not alone responsible for all the good or evil in the world, but it is more conducive to such results than any other factor. In agriculture, take the best of seed planted in soil rich with the elements best adapted to the variety of seed, and with proper care and attention during its growth a well developed, abundant harvest will be reaped.

So horse-breeders develop fine blooded stock with peculiar characteristics, such as race-horses, trotters, roadsters, and draft-horses, at will. In the canine, the spaniel, watch-dog, pug, rat-terrier, etc., are thus produced.

This principle of heredity is plainly manifest all through the vegetable as well as the animal kingdom, and is capable of elevating or degrading the race in the same proportions as its principles are recognized and put in practice.

*Criminals are thus perpetuated and crime increased, while parents with such propensities transmit them to their offspring and increase such proclivities by training and association.*

*Such people are not fit subjects to procreate, and should be controlled by law for their own sakes,* the sake of the community in which they live, for the advancement of morality, virtue, and Christianity.

Such persons should not be allowed to bring children into the world to suffer, be disgraced, degraded, and cause other persons to suffer. *Certain crimes should render the perpetrator amenable to punishment by rendering them sterile or by isolation for life.* By so doing they would not be allowed to propagate, causing degradation, misery, shame, and ruin, not only to their own offspring, but to the community in which they live, causing innocent ones to suffer, and developing a power for evil by association and influence. A bad boy can contaminate a whole community, and a bad man has influence in the same proportion. Innocent, unsuspecting victims are frequently by such led to ruin and despair.

They ruin both soul and body, render life miserable, destroy hope beyond that veil "whose green curtain never outward flows."

Such depraved beings have no right morally, and should not have legally, to propagate their vices.

We as law-abiding, liberty-loving citizens, aid and abet such by our indifference and want of action in many instances, and in others license evil business and influences, when we know their results are evil, and innocent, helpless victims are made to suffer. I regret to say that such business and evil influences are frequently licensed by men, or through the influence of men, who claim to be moral, upright, law-abiding, honorable citizens, simply for the pitiful handful of "filthy lucre" that the license brings. Selling souls, morality, virtue, not so much of themselves individually, but their neighbors, their neighbors' children, aye, often their own children or grandchildren, and of countless innocent babes yet unborn.

Who can count the cost of such licensed evil influences, financially, socially, morally, or religiously? None but an infinite, all-wise mind. Of all things in this world, give us a few generations of men and women, not fickle, one-sided, weakly, puny human beings, willing to sacrifice the morals and virtue of their neighbors and neighbors' children, yea, even their own, for "filthy lucre" and the temporary gratification of their own lusts, ambitions, and appetites, but men and women honest, upright, and conscientious in all things, whose thoughts, actions, and deeds are pure, and then we would have a race of people a pleasuse to behold.

Such a condition is possible, but cannot be reached when the leaders in state, religion, and business are immoral and impure.

Parents transmit that which they are and possess. If their thoughts, actions, deeds, and very make-up are evil, then it will manifest itself in their offspring.

Clear, pure, refined water does not flow from a mudhole, but if the fountain is pure, from it pure, crystal water will flow. If you desire pure, honest, upright, loyal children, be so yourself, then you not only transmit such traits, but elevate them by precept and example.

Space forbids further thoughts in this line, but we cannot refrain from urging that transmitting certain diseased conditions should be prohibited by law.

Gonorrhea has ruined many a wife and sacrificed the eyes of many children by ophthalmia neonatorum because of the sins of the parents, especially the father. "Syphilis has slain its thousands," poisoning the life-blood of the innocent and pure simply for the gratification of lust. No man or woman suffering with either syphilis or gonorrhea should be allowed to take upon themselves the bonds of matrimony until the disease has been thoroughly eradicated by treatment. It is here the innocent and helpless ones are made to suffer for the sins of the guilty, which could and should be prevented.

As to consumption, insanity, and other hereditary diseases, it is a more difficult question to decide. It would be best for persons with a predisposition to such diseases not to marry, not only for their own good, but for the good of humanity and the physical development of the race. These conditions are not brought on by a sin of the individual, as in venereal disease, but are received by transmission. The noblest, purest person may thus be afflicted. Even then, where there is a marked predisposition to such disease in the family, they should forego the pleasures of married life for the sake of humanity and the lessening of human suffering. Such sacrifices would be humane, honorable, heroic, and should be encouraged and commended by all right-minded people. The human race will never reach that high plane of physical, moral and spiritual development that it should so long as wickedness is winked at in high places, criminals and transgressors are allowed to propagate their vices *ad libitum*, lust goes unrestrained, and matrimony consummated simply by blinded love without regard to fitness, morality, mentality, or diseased conditions. Sensible men and women frequently select life companions with less thought as to fitness, qualifications, hereditary tendencies, mental qualities, family history and characteristics than the fancier selects his pigeons, dogs, or horses; even in most instances with less investigation than even the horse jockey trades horses.

True love, guided by proper judgment and discretion, is a boon to humanity, but sentimentalism and blinded passion has no place in the selection of a companion. After wedlock the true character of each is displayed. Then it is sentimental love vanishes and each are brought face to face with the realities of life and the imperfections of each are made manifest. Then the battle of life comes, in adapting and changing the habits, tastes, and mentality of one or both so as to harmonize with their environments.

Many a useful life, and would-be happy home, is wrecked near the shore before deep water is reached for smooth sailing. Does the farmer select for sowing smutty wheat, shriveled grains, or seed mixed with cheat, and cast it upon poor barren, rocky, unprepared soil? If so, what will he reap?

Can you harmonize the tastes, habits, and customs of the rat terrier with the shepherd dog, the poodle with the bloodhound, the pug with that of a setter? To produce a roadster, trotter, or race-horse would you select as parent a dray-horse or Shetland pony? If men and women would exercise more judgment and discretion in selection of life companions, at least display as much thought and caution as in other avenues of business, the human race would be greatly benefited and divorce suits less frequent.

When properly considered, there is no responsibility so great in the whole range of human life as that of selecting a husband or a wife—a union for life and the creating or developing of human souls bearing the impress of your individuality and habits—souls that may be a benefaction or curse to humanity, a pleasure and a delight, or a curse and a disgrace to an enlightened civilization. If you want crime and degradation as a predominating factor in your children, marry into a family of criminals, select from the gamblers' host, or choose from a family of drunkards; but if you prefer the opposite, choose otherwise. Marriage is not a failure, but wonderfully abused. Properly consummated, it is the corner-stone of true happiness, the golden chain that forms the strongest ties and the greatest power we have for the purification of the human race. Where it fails the individuals are at fault and not the institution of marriage. A congenial union in the bonds of matrimony stimulates the development of true manhood, enures to higher aims and nobler aspirations in life, and lessens vice and immorality. There is no human power greater for good than a pure, devoted, true mother, and a bad one is just as potent for evil. Mothers bear a double part in the development of the human race, which fact, in a measure, has been the redemption of mankind, for women, as a class, are far more moral than men.

Very few women are gamblers, criminals, swear, drink, use tobacco, or practice lewdness, which cannot be said of men. There should be the same standard of morals for man as for woman; then the physical, moral, and intellectual tone of mankind would be rapidly elevated. Selfish man gratifies his own lusts and sinful pleasures, but denys his self-sacrificing wife the same privileges. They must not only deny themselves sinful indulgences, but often the legitimate pleasures and even the necessities of life. The first a blessing in disguise, the latter unjustly forced upon them. If this were all it would not be so bad, but they are compelled, as a dutiful, noble wife, to bear children, tainted with the physical habits and mental characteristics of the father by heredity, and later the child is influenced by training and association, precept and example of the father, but thanks be to the noble mother, the child is often redeemed by her good qualities. Nature has well provided that the mother shall not only furnish her own part of the germ life, but contribute far greater to the development of the child, not only during utero-gestation, but by closer association during the formative period of the child after birth. Thus it is she has the greater power for good or evil, and we are glad to say it is usually exerted for good, and would be used to a far greater advantage if they more fully understood the possibilities that exist in this line for improvement of the race. The boys and girls of to-day will be the fathers and mothers of the next generation. Are they readers of "sensational novels," "blood and thunder stories," card-players, drinkers of the social wine cup; if so, the father's grey hairs will go to the grave in sorrow, the mother's life will be shortened by grief, and a blind man can prophesy the fate of the next generation. If the girl is raised a sickly, puny, fickle, finicky, nervous creature, with depraved appetites, constricted waist, dwarfed physically, mentally and intellectually, is she a fit subject to become a mother?

Space in this article forbids vital and important details of thoughts and suggestions on the development of the girl and on training and association as primal factors in the improvement of the human race.

The mothers of to-day have many imperfections, and infirmities, especially in city life, brought about by heredity, imperfect development, faulty training, fast living, and the demands of fashionable society. She is ushered into matrimony and wafted into maternity with but little knowledge of her physiological functions and duties for either, an ignorance which has sacrificed the health and lives of many.

Physiologically the mother furnishes her part of the germ life (ovule), the entire pabulum and environment for the nine months of the utero-gestation, and the greater part afterward, as a rule, during lactation (nursing period). Thus it is she contributes more to the welfare and development of the child than the father. Hence the necessity that she be healthy, well developed, physically, mentally, and morally.

There is no doubt but that the fetus in utero is influenced in its development by conditions of the mother during pregnancy. It is also pretty well established that *prolonged agreeable mental thought*, study, and investigation in certain lines by the mother during gestation will transmit a certain tendency or trend in the child to pursue the same line of thought or investigation.

Observe, that we say prolonged *agreeable* mental thought or study, not a line of thought or study repulsive to the mother, but something in which she can take pleasure and delight for the benefit of her offspring. It should not be a transient, changeable line of thought, nor one far beyond the mentality of the mother to comprehend; neither should it be so assiduously or closely pursued as to tax the mental or vital powers of the mother to the exclusion of physical exercise and the minor details that are essential to the establishment of her normal healthy functions, including a proper moral and spiritual development.

The father or mother should not be extremists in morals or religion, else by their very overzealousness they may consume all their vital powers in that direction, leaving but little to be transmitted to their offspring; besides, extreme views are liable to become repulsive to the young and create an indifference to the principles which it is specially desired to inculcate. Firmness and stability of character, with adherence to right principles and truth, should be cultivated in all and developed in the offspring. The transmission of these mental characteristics, with proper hereditary development and after-training, are wonderful factors in the development of intellectual, moral and upright men and women.

A potent factor for evil is the habitual use of stimulants by the father or mother. I quote the following, which speaks for itself:

"Professor Belman, of the University of Bonn, in *Medical Press*, relates the career of a notorious drunkard, who was born in 1740 and died in 1800. Her descendants numbered 834, of whom 709 have been traced from their youth. Of these, 7 were convicted of murder, 76 of other crimes, 142 were professional beggars, 64

lived on charity, and 181 women of the family led disreputable lives.

"The family cost the German government for maintenance and costs in courts, almshouses, and prisons no less a sum than $1,250,-000—in other words, just a fraction under $1,500 each."

The habit of some mothers, of their own accord, or even by the advice of a physician, of drinking wines, whisky or beer during pregnancy or while nursing, is to be condemned, as it fosters intemperance and tends to develop habits and appetites in the mother, as well as in the child, at a formative stage in its growth and development, to bear fruit in later years.

The conscientious physician should think seriously before advising such treatment of pregnant or nursing women.

If the mother is diseased, the physician should seek the *cause* and *remove it*, but not *advise the use of alcoholics as a cloak for his ignorance, when it may be followed by evil consequences to both mother and child. If she is diseased, she needs treatment, not alcoholics, as they cure but few diseases*, in most cases at best are palliative only, and frequently injurious. Even at the risk of being called an "extremist" by some unthinking people or those that indulge in its use, I would include tobacco as frequently injurious, and when habitually used by father and mother, will transmit tendencies and appetites which they have no moral right to do simply to gratify indulgence in a luxury.

While "maternal impressions," "mother's mark," etc., as recognized by the general public, are mere speculation and without foundation, yet embryologists are not prepared to deny the possibility of "maternal impressions" occurring in the early months (first three or four) of gestation. The far greater majority of malformations or deformities that occur are due to arrest or want of development, diseased conditions for causes inherent to the ovum itself and not due to any transient impression, nervous shock or scare of the mother.

It is self-evident that for the best interests of the child the mother, during gestation, should avoid *prolonged* mental worry, anger, or unpleasant thoughts; that her physical condition be kept in the best state possible, the mind cheerful and occupied in pleasant lines of thought, which will have a tendency to elevate rather than degrade the offspring.

Fretful, peevish, ill-tempered children are frequently but the counterpart of the mother, else diseased or simply "growing more like their father every day." They are simply a reproduction of the parents, inheriting their good or bad qualities according as such traits predominate in the parents.

A wise man has said, "Train up a child in the way he should go, and when he is old he will not depart from it." This is a maxim every parent should remember, and fulfill its obligations.

Many a child is spoiled in its raising, although it may have been born with the greatest possibilities for good. Many are never taught obedience, respect to law, nor to consider the rights of others while young, disobeying parents, thus reaching maturity with proclivities for disobedience, increasing as they grow older; later are law-breakers, trespassers on the moral rights of others, and lending an influence for sinful indulgences.

To attain that for which the human race was created demands purity of thought, purity of action, purity in words, purity in all physiological functions. Without either no man or woman is complete. Pure water cannot flow from a mud-hole, a stagnant pond, nor the ocean. Neither can pure children come from the filth, squalor, and slums of the city, nor from that more respectable class of gamblers and drinkers who are actuated by sinful indulgences and lewdness, no, not even from the aristocracy that revel in luxury, wealth, and *sinful pleasures* unless they be purified by a power not inherent to themselves.

It is a law more unchangeable than that of the Medes and Persians, that a thing cannot transmit that which it does not possess.

Then will *you* transmit as a legacy to your children the *possibilities* of the greatest good, developing a pure, good man or woman; or will you, by your own free will and indulgences, transmit and develop in *your own child* the possibilities for evil, degradation, and ruin which does not stop with the child, but is visited even to the third and fourth generations after you? The nearer we reach perfection physically, morally, and mentally, the less disease and suffering we have.

An immoral and corrupt people will become diseased and degraded physically, while a moral, upright people will be improved thereby.

Morality and uprightness tend to prevent disease and favor physical development, while sin and immorality tend to produce disease and degrade physically. A woman properly developed, physically, morally and mentally, is well equipped for matrimony, and can fulfill the functions of maternity with grace and dignity, with little suffering, few diseases and accidents.

The prevention of conception and induction of abortion are becoming potent factors for evil, especially in city life. Their practice degrades morally except where legitimate diseased conditions demand their use. The devices used to close or cover mouth of the womb obstruct drainage and produce disease, while the use of cold water is more dangerous, often menacing the life of the user. Producing abortion is more dangerous and more immoral, often resulting in the death of the mother. It is simply the taking of human life—a sin against humanity, morality, and Christianity, deserving of censure and condemnation by all right-minded people.

As soon as conception takes place life begins, and he who destroys it at three weeks or three months is equally as responsible as the one who destroys it at six or nine months.

We can not help looking to the future with fear and trembling when so many of our women, seemingly of good moral character, resort to such measures and manifest no compunctions of conscience for their sin against God and humanity. Equally strange is it that the professional abortionist can ply his or her nefarious work and prosper in a Christian, moral community without being called to account for violation of law and moral decency.

Such influences as the above seem to manifest themselves in the medical profession by developing a too frequent tendency to empty the pregnant uterus under the plea that "such action is necessary to save the life of the patient."

Producing abortion should carry with it a great weight of responsibility not only from a legal and moral standpoint, but from the dangers inherent to the operation itself, especially when not done in a clean aseptic manner. Most women fail to recognize the danger to life and the frequency with which diseased uteri, tubes and ovaries are produced by induced abortions, or abortions improperly performed or treated by incompetent persons.

Diseases are thus produced which may result in permanent impairment of tubes and ovaries and increase of the dangers incident to labor at full term. Induced abortions, improper treatment, or handling of cases of labor and abortions frequently develop diseased conditions which render subsequent labors more dangerous and liable to complications.

(*To be continued.*)

